# Update Amputationsregister Deutschland (AMP-Register)

**DOI:** 10.1007/s00113-025-01539-0

**Published:** 2025-02-13

**Authors:** Merkur Alimusaj, Kristina Michel, Julia Block, Urban Daub, Daniel Heitzmann, Thanh-Duc Nguyen, Maria Bisele, Sebastian I. Wolf, Urs Schneider

**Affiliations:** 1 Zentrum für Orthopädie, Unfallchirurgie und Paraplegiologie, Universitätsklinikum Heidelberg, Schlierbacher Landstr. 200a, 69118 Heidelberg, Deutschland; 2 Fraunhofer-Institut für Produktionstechnik und Automatisierung IPA, Stuttgart, Deutschland; 3 MeTKo-Zentrum, Heidelberg und Stuttgart, Deutschland

**Keywords:** Registerforschung, Bein, Prothese, Technische Orthopädie, Orthopädietechnik, Register-based research, Lower limb, Prosthesis, Technical orthopedics, Orthopedic techniques

## Abstract

**Hintergrund:**

In medizinischen Registern werden wertvolle Daten von Patienten gesammelt, die die Qualität und Wirksamkeit von Behandlungen überprüfen bzw. kontrollieren. Es existieren nationale Register im Bereich der Amputationsmedizin und Patientenversorgung wie das Swedish Amputation and Prosthetics Registry (SwedeAmp) und das Limb Loss and Preservation Registry (LLPR) in den USA, die Informationen zu Prothesenarten, -materialien und -verfahren sowie patientenbezogene Ergebnisse wie Mobilität und Lebensqualität erheben. Seit 2011 konnte SwedeAmp wichtige Erkenntnisse zu Langzeitergebnissen nach Amputationen beitragen und die Versorgung in Schweden verbessern. Das LLPR in den USA erfasst Daten von klinischen bis zu psychosozialen Aspekten, was länderübergreifende Vergleiche und die Optimierung der Versorgung ermöglicht.

**Material und Methoden:**

In Deutschland leistet das AMP-Register bedeutende Beiträge, indem es u. a. Daten zu Prothesenpassform und -tragekomfort sowie Gründe für Revisionen dokumentiert. Ziel ist es, eine Evidenzbasis durch systematische Datenerhebung zu schaffen. Das Projekt umfasst den Aufbau einer benutzerfreundlichen IT-Struktur, eine Pilotphase zur Anwendungsevaluierung und die enge Zusammenarbeit mit Experten. Mithilfe standardisierter Datensätze sollen Versorgungsdefizite aufgedeckt und evidenzbasierte Ansätze entwickelt werden. Datenerfassung und -speicherung erfolgen gemäß der Datenschutz-Grundverordnung (DSGVO) und werden durch technische Maßnahmen abgesichert.

**Ergebnisse und Diskussion:**

Erste Ergebnisse am Studienzentrum Heidelberg zeigen das Potenzial des AMP-Registers. Subgruppenanalysen unterstützen die Versorgungsoptimierung und bestätigen die Relevanz regelmäßiger Assessments, um die Versorgungsqualität langfristig zu verbessern.

## Abstract (en)

**Background:**

Medical registries are used to collect valuable patient data in order to check and control the quality and effectiveness of treatment approaches. National registries in the field of amputation and patient care, such as the Swedish Amputation and Prosthetics Registry (SwedeAmp) and the Limb Loss and Preservation Registry (LLPR) in the USA, gather information on prosthetic devices, materials, procedures and patient-related outcomes such as mobility and quality of life. Since 2011 SwedeAmp could generate significant insights into long-term outcomes after amputations and improve prosthetic care in Sweden. In the USA the LLPR collects data on aspects ranging from clinical to psychosocial aspects, enabling international comparisons and the optimization of care.

**Material and methods:**

In Germany the AMP Registry makes significant contributions by documenting data on prosthetic fit, wearing comfort and the reasons for revisions. The aim of the AMP Registry is to create an evidence base through systematic data collection. The project includes the development of a user-friendly IT structure, a pilot phase for application evaluation and close collaboration with experts. Standardized datasets aim to identify deficits in care and develop evidence-based approaches. Data collection and storage are carried out in compliance with the General Data Protection Regulation (DSGVO) and secured through technical measures.

**Results and discussion:**

Initial results from the Heidelberg Study Center demonstrate the potential of the AMP Registry. Subgroup analyses support optimization of care and highlight the importance of regular assessments to improve long-term care.

In Deutschland werden jährlich etwa 60.000 Amputationen durchgeführt [12–14]. Die Versorgung dieser Patienten erfordert eine strukturierte Betreuung zur Wiederherstellung der Mobilität mit Prothesen. Intersektorale und interdisziplinäre Hürden führen oft zu Informationsverlusten zwischen stationärer und ambulanter Versorgung sowie den Rehabilitationsphasen, was sich negativ auf die Patientenerfahrung auswirkt und den Austausch der Akteure erschwert [3–5, 12]. Die aktuelle europäische Verordnung zu Medizinprodukten, die sogenannte Medical Device Regulation (MDR) erhöht die Anforderungen an die klinische Bewertung und Sicherheit von Sonderanfertigungen wie Prothesen, ohne standardisierte Methoden bereitzustellen. Register in der Beinprothetik liefern wichtige Daten zur Verbesserung von Behandlungsqualität und -effektivität [7]. Solche Register erfassen Daten zu Prothesentypen, -materialien und chirurgischen Verfahren sowie zu patientenbezogenen Ergebnissen wie Mobilität und Lebensqualität. Diese ermöglichen die Evaluierung der Versorgungsqualität und die Entwicklung evidenzbasierter Standards. In vielen Ländern existieren nationale Register mit spezifischen Schwerpunkten zur Datensammlung.

## Hintergrund

Das Swedish Amputation and Prosthetics Registry (SwedeAmp) sammelt seit 2011 Daten zur Versorgung von Menschen mit Amputationen in Schweden. Neben demografischen Angaben und Amputationsgründen erfasst SwedeAmp auch Prothesenarten, postoperative Komplikationen und Revisionsraten. Besonders wertvoll ist die systematische Erfassung von funktionellen Ergebnissen und Lebensqualität, die Aufschluss über die Effektivität und Patientenzufriedenheit geben. SwedeAmp hat in der Forschung wichtige Erkenntnisse zu Langzeitergebnissen und Einflussfaktoren auf die Prothesennutzung geliefert; diese haben die Versorgungsqualität in Schweden verbessert [7].

Das Limb Loss and Preservation Registry (LLPR) in den USA verfolgt ähnliche Ziele wie das AMP-Register. Es sammelt standardisierte Daten zu Amputationen und Prothesenversorgungen, einschließlich demografischer, klinischer, funktioneller und psychosozialer Aspekte. Das LLPR liefert wertvolle Erkenntnisse über die Auswirkungen von Komorbiditäten und regionalen Versorgungsunterschieden auf die Prothesenanpassung sowie den Erfolg der Versorgung. Zudem fördert es die Verbesserung der Versorgung durch länderübergreifende Vergleiche und dient oft als Vorbild für internationale Register [8].

Das deutsche AMP-Register trägt wesentlich zur Amputationsprothetikforschung bei, indem es Daten zu Beinprothesen sowie deren medizinischen und funktionellen Ergebnissen, einschließlich Passform, Tragekomfort und Wechselgründen, sammelt. Putz et al. [11] betonen in ihrer Veröffentlichung die Bedeutung der systematischen Datenerfassung für Qualitätsverbesserungen sowie die Identifikation von Trends zur Optimierung von Prothesenanpassung und -haltbarkeit.

Register wie SwedeAmp, LLPR und das deutsche AMP-Register fördern Qualitätssicherung, Benchmarking und die Verbesserung der Amputationsversorgung. Sie ermöglichen Kliniken, Versorgungsstrategien zu optimieren, und unterstützen evidenzbasierte Empfehlungen. Internationale Kooperationen mit Registerdaten helfen, globale Standards und Best Practices zu entwickeln [10].

Register fördern Qualitätssicherung, Transparenz die Verbesserung der Amputationsversorgung

Zusammenfassend lässt sich festhalten, dass Register wie SwedeAmp, LLPR und das AMP-Register Deutschland nicht zu unterschätzende und wesentliche Instrumente in der Beinprothetik sind. Sie liefern wertvolle Daten zur Versorgungsqualität und unterstützen die Entwicklung von Standards zur Verbesserung der Patientenversorgung. Solche Register fördern zudem die Evidenzbasis, auf der moderne Behandlungsansätze in der Prothetik aufbauen und schaffen eine transparente Grundlage für die Weiterentwicklung der Prothetiktechnologie und der Rehabilitation.

## Projektübersicht

### Ziel

Das Ziel des Projekts AMP-Register ist die Entwicklung eines deutschlandweiten Registers zur Verbesserung der Evidenzbasis in der Versorgung von Menschen nach einer Beinamputation. Es wird vom Medizinisch-Technischen Kompetenzzentrum für Orthopädietechnik (MeTKo), einer Kooperation der Orthopädischen Universitätsklinik Heidelberg und dem Fraunhofer-Institut für Produktionstechnik und Automatisierung (IPA), geleitet und verfolgt folgende Ziele:

- systematische Erfassung patientenbezogener Daten,
- Entwicklung einer benutzerfreundlichen IT-Struktur,
- Implementierung einer Nutzerverwaltung zur Administration beim Register-Partner,
- Sicherstellung langfristiger Nutzung durch rechtliche und geschäftliche Lösungen,
- Pilotphase zur Prüfung der Anwendbarkeit,
- Einbindung relevanter Stakeholder.

### Grundlagen

Die Datenstruktur wurde basierend auf eigenen Vorarbeiten in Zusammenarbeit mit der Deutschen Gesellschaft für interprofessionelle Hilfsmittelversorgung e. V. (DGIHV) und dem Bundesverband für Menschen mit Arm- oder Beinamputation e. V. (BMAB) weiterentwickelt [2], um in der Konsequenz, auf standardisierten und anerkannten Daten fußend, langfristige Analysen und Vergleiche zu erlauben. Eine IT-Struktur wurde geschaffen, um auch verschiedene Nutzungsszenarien, wie beispielsweise einen Wechsel des Leistungserbringers, zu berücksichtigen. Eine grafische Darstellung optimierte die Nutzererfahrung.

Zur nachhaltigen Anwendung wurde ein Geschäftsmodell entwickelt, einschließlich Schulungsprogrammen zur Nutzung der App AMP-Kompass. Erste Schulungen, auch an Berufsschulen, waren erfolgreich.

In der Pilotphase sammelten nach ersten Vortests an der Technischen Orthopädie des Universitätsklinikums Heidelberg Sieben weitere Leistungserbringer und Zentren in Baden-Württemberg erste Daten. Die Netzwerkarbeit, z. B. auf der OT-World in Leipzig, förderte Kooperationen und die Weiterentwicklung des Registers, um internationale Best Practices zu integrieren.

Der konkreten Entwicklung des Registers gingen mehrjährige Arbeiten, die zunächst den Grundstein legten, voraus. Wesentlich war das vom Forum Gesundheitsstandort Baden-Württemberg (Ministerium für Soziales und Integration Baden-Württemberg) geförderte Vorprojekt AMP-Kompass mit folgenden Inhalten:

1. Etablierung der standardisierten Profilerhebung und erfolgreiche Anwendungen im klinischen Umfeld,
2. Implementierung einer Infrastruktur zur dezentralen Nutzer- und Rechteverwaltung,
3. Pilotphase mit positiven Ergebnissen zur App-Nutzung und Feedback zur weiteren Optimierung,
4. Netzwerkbildung mit wichtigen Akteuren aus dem Gesundheitswesen, einschließlich der Orthopädietechnik.

Das Projekt zeigt erste Erfolge in der Umsetzung eines Registers für die Versorgung von Menschen nach Beinamputationen. Die Infrastruktur und das Schulungskonzept stehen, und die Pilotphase läuft vielversprechend. Die Weiterentwicklung wird sich auf die Optimierung der Nutzererfahrung und die Ausweitung auf weitere Register-Partner konzentrieren. Auch die Schaffung eines nachhaltigen Geschäftsmodells sowie die Sicherstellung der Datenintegrität und -sicherheit sind priorisierte Ziele.

### Datenschutz und Auftragsverarbeitung

Ein zentraler Aspekt der Registerarbeit ist der Aufbau einer datenschutzkonformen Struktur. Dazu gehören Vereinbarungen zur Datenverarbeitung mit allen Register-Partnern. Ein Kooperationsvertrag zwischen dem Universitätsklinikum Heidelberg, dem Fraunhofer IPA und den klinischen Partnern regelt die Zusammenarbeit und Nutzung der Registerdatenstruktur. Ergänzend dazu legt eine an den Vertrag angehängte Vereinbarung, gemäß Art. 26 der Datenschutz-Grundverordnung (DSGVO), die Pflichten und die gemeinsame Verantwortung bei der Verarbeitung personenbezogener Daten fest.

### Datenfluss und Datenstromkonzept

**Abb. 1:**
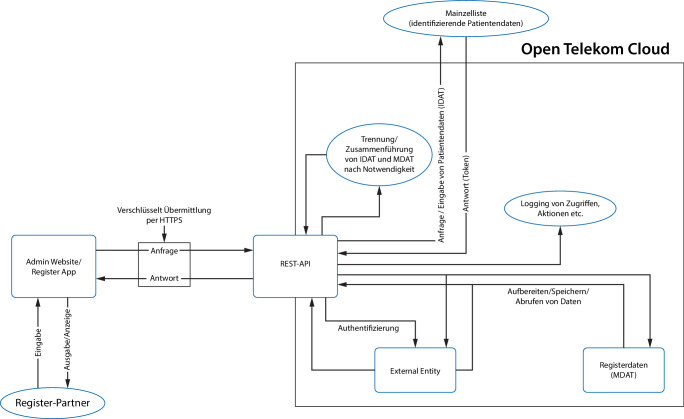
Datenstromkonzept des AMP-Registers. *HTTPS* Hypertext Transfer Protocol Secure, *IDAT* identifizierende Daten, *MDAT* medizinische Daten, *REST-API* (Representational State Transfer Application Programming Interface) als Schnittestelle

Die Abb. 1 zeigt eine schematische Darstellung des Datenstromkonzepts von AMP-Register. Die gezielte Steuerung der Datenflüsse ermöglicht eine sichere Erfassung und Verwaltung von Patientendaten und basiert auf mehreren zentralen Komponenten.

Die Open Telekom Cloud ist die zentrale Infrastruktur für Datenspeicherung, Benutzerverwaltung und Authentifizierung. Die Datenbank ist in medizinische Daten (MDAT) und identifizierende Daten (IDAT) unterteilt.

Medizinische Daten werden in einer separaten, speziell dafür entwickelten Datenbank gespeichert. Die Trennung von identifizierenden und medizinischen Daten erfolgt durch die Mainzelliste als Datentreuhänder [9]. Die Mainzelliste sorgt für die Pseudonymisierung und sichere Verwaltung der identifizierenden Daten, während medizinische Daten getrennt verarbeitet werden. Benutzerdaten enthalten Authentifizierungsinformationen, Logins, Zugriffe und Aktionen.

Die Architektur des AMP-Registers gewährleistet dessen sicheren und effizienten Betrieb

Eine REST-API (Representational State Transfer Application Programming Interface) verbindet Systemkomponenten und ermöglicht die flexible Zusammenführung oder Trennung von IDAT und MDAT. Zusätzliche Systeme außerhalb der Open Telekom Cloud umfassen eine Admin-Website und einen Webserver für Benutzer- und Systemverwaltung mit verschlüsselter REST-API-Kommunikation. Eine mobile App ermöglicht Patienten und Experten Dateneingabe und -anzeige sowie Datenverwaltung und -visualisierung. Die Architektur gewährleistet den sicheren und effizienten Betrieb des AMP-Registers durch Trennung und Pseudonymisierung persönlicher und medizinischer Daten sowie eine zentrale Cloud-Infrastruktur.

### Erste Ergebnisse

Die Analyse der ersten Datensätze der vorgelagerten monozentrischen Testphase am Studienzentrum Heidelberg zeigt das Potenzial einer strukturierten Dokumentation. Von 712 erhobenen Datensätzen wurden 498 für die erste Auswertung berücksichtigt (Tab. 1).

**Tab. 1:** Beschreibung der Stichprobe von Patienten nach einer Beinamputation

Merkmale	Ausprägung
Stichprobengröße (Anzahl, *n*)	498
Alter (Jahre, Median [Min–Max])	57 (4–93)
Geschlecht (männlich/weiblich)	337/159
Körpergröße (cm, Median [Min–Max])	175 (55–198)
Körpergewicht (kg, Median [Min–Max])	78 (11–189)
Zeit seit der Amputation (Jahre, Median [Min–Max])	4,1 (0,1–72)

Die analysierte Stichprobe weist ein Durchschnittsalter von 57 Jahren auf und umfasst Betroffene zwischen 4 und 93 Jahren. Männer sind mit 337 gegenüber 159 Frauen in der Stichprobe häufiger vertreten. Die Körpergröße beträgt im Durchschnitt 175 cm, wobei insgesamt eine relativ große Bandbreite von 55–198 cm abgedeckt wird. Das Körpergewicht beträgt im Mittel 78 kg; die leichteste Person hat 11 kg und die schwerste 189 kg gewogen. Der Zeitraum seit der Amputation reicht von wenigen Monaten bis zu 72 Jahren.

Hauptamputationsursachen sind v. a. traumatische Ereignisse

**Abb. 2:**
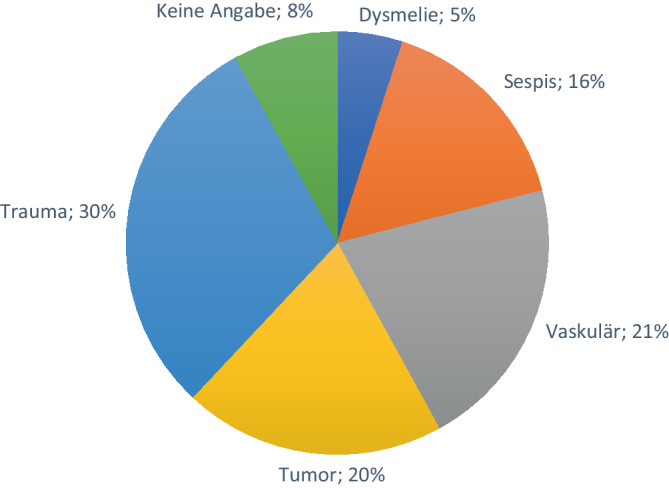
Verteilung der Amputationsursachen

In der ersten Auswertung wurden diverse Parameter näher betrachtet bzw. ausgewertet. So wurde überprüft, welche Ursachen für die Amputation bei den erfassten 498 Personen vorlagen. In Abb. 2 ist die prozentuale Verteilung der Amputationsursache dargestellt. Die Gesamtheit der genannten Ursachen waren Dysmelie, Sepsis, Tumor, vaskuläre Gründe oder ein Trauma, wobei von 8 % der 498 Personen keine Angabe gemacht wurde. Die Hauptamputationsursachen sind mit 30 % v. a. traumatische Ereignisse, gefolgt von Tumorerkrankungen (20 %), was sich auch in der Sprechstunde der Klinik als offenbarer Standortfaktor widerspiegelt (Abb. 2).

Mit 21 % sind vaskuläre Gründe eine weitere, häufig auftretende Ursache für eine Amputation. In der vorgestellten Stichprobe aus dem Studienzentrum Heidelberg zeigt sich, dass im Gegensatz zum Bundesdurchschnitt der Grund „vaskulär“ nicht als vermutete Hauptamputationsursache zutrifft. Es ist zu erkennen, dass etwa 79 % der Stichprobe nichtvaskulär bedingt amputiert worden sind.

Ein Großteil der Patienten (75 %) hatte zum Zeitpunkt des Einschlusses in das AMP-Register bereits eine prothetische Versorgung erhalten. Zum Zeitpunkt der Erhebung nutzten 54 % der Betroffenen aktiv eine Prothese, 18 % nutzen sie eingeschränkt, und 13 % verwendeten die vorhandene Prothese aktuell nicht (Tab. 2).

**Tab. 2:** Angaben zu Therapie und Versorgung

*Hatten Sie bereits eine prothetische Versorgung?*	
Ja	288 (75 %)
Nein	74 (19 %)
k. A.	24 (6 %)
Anzahl (*n*)	386
*Verwenden Sie derzeit eine Prothese?*	
Ja	210 (54 %)
Eingeschränkt	71 (18 %)
Nein, gar nicht	51 (13 %)
k. A.	54 (14 %)
Anzahl (*n*)	386

*k.* *A.* keine Angabe

Patienten, die ihre Prothese derzeit nicht oder nur eingeschränkt nutzen, lassen sich weiter analysieren. Unter ihnen befinden sich beispielsweise Patienten nach chirurgischen Revisionen.

Es wurde auch erhoben, ob eine Gehschule Teil der prothetischen Versorgung war, was von 40 % der befragten Personen bejaht wurde. Etwa 92 % der Teilnehmenden überwinden täglich Treppenstufen, 53 % und 47 % begeben sich mehrmals wöchentlich auf unebenes Gelände bzw. überwinden Steigungen. Zudem gaben 34 % der Betroffenen an, in den letzten 4 Wochen gestolpert zu sein, wobei 16 % sogar gestürzt waren (Tab. 3).

**Tab. 3:** Angaben zur Nutzung der Prothese im Alltag und zu ihrer Vorbereitung im Rahmen der Rehabilitation

*Ist im Rahmen der prothetischen Versorgung jemals eine Gehschule erfolgt?*	
Ja	40 %
Nein	60 %
Anzahl (*n*)	251
*Müssen Sie täglich Treppenstufen überwinden?*	
Ja	92 %
Nein	8 %
Anzahl (*n*)	368
*Gehen Sie derzeit mehrmals wöchentlich auf unebenem Gelände?*	
Ja	53 %
Nein	47 %
Anzahl (*n*)	310
*Gehen Sie derzeit mehrmals wöchentlich auf Schrägen und Rampen?*	
Ja	47 %
Nein	53 %
Anzahl (*n*)	306
*Sind sie innerhalb der letzten 4 Wochen gestolpert/gestürzt?*	
Ja	34 %/16 %
Nein	66 %/84 %
Anzahl (*n*)	306/306

Menschen mit Fußamputationen erhalten oft keine adäquate prothetische Versorgung

Weitere Subgruppenanalysen (nicht grafisch abgebildet) zeigten beispielsweise, dass Menschen mit Fußamputationen oft keine adäquate prothetische Versorgung erhalten und auf provisorische Hilfsmittel angewiesen sind. Eine weitere Untersuchung von Patienten, die aufgrund von Tumoren oder Traumata amputiert wurden, ergab, dass diese im Durchschnitt jünger sind und ein aktiveres Leben führen als Patienten, die aufgrund vaskulärer Störungen amputiert worden waren.

#### Infobox Weiterführende Informationen

Mehr Informationen zum Thema: https://metko-zentrum.de/

Profilerhebungsbogen über: www.dgihv.de

Für Rückfragen: AMP-Register.OUK@med.uni-heidelberg.de

## Zukunftsperspektiven

Die jährlich erhobenen Daten dienen als Grundlage zur Bewertung der Amputationsversorgung und werden teilweise im Rahmenvertrag der AOK Baden-Württemberg und des Fachverbands für Orthopädietechnik genutzt [2]. Die bisherigen Ergebnisse zeigen das Potenzial strukturierter Dokumentation für individuelle Verläufe und die Analyse von Versorgungsstrukturen [15]. Die digitale Erhebung läuft erfolgreich in Baden-Württemberg, und Kliniken äußerten Interesse an der Verbesserung der Versorgungsqualität. Erste Erkenntnisse aus dem AMP-Register und aus internationalen Registern belegen die Vorteile solcher Strukturen für die Versorgung.

Die Zukunft der Hilfsmittelversorgung wird durch systematische Untersuchungen und Assessments geprägt

Das Projekt dient auch als Vorlage für andere Bereiche der Technischen Orthopädie zur Schaffung einer praktikablen und alltagstauglichen digitalen Datenerfassung innerhalb des Versorgungsprozesseses. Besonders bei individuell angepassten Hilfsmitteln können resultierende evidenzbasierte Empfehlungen Effizienz und Mehrwert steigern. Die Zukunft der Hilfsmittelversorgung wird durch systematische Untersuchungen und Assessments geprägt [1]. Diese Werkzeuge ermöglichen zudem eine präzise Einschätzung individueller Bedürfnisse und Fähigkeiten, verbessern die datengestützte Versorgung und steigern Funktionalität, Nutzen der Hilfsmittel und die Lebensqualität der Betroffenen.

### Stellenwert der klinischen Untersuchungen für die personalisierte Versorgung

Klinische Untersuchungen erfassen den Gesundheitszustand, die Mobilität und Bewegungsfähigkeit von Patienten. Tests zu Muskelkraft, Gelenkfunktion und Schmerzempfindlichkeit liefern entscheidende Daten für die passgenaue Anpassung von Prothesen oder Orthesen. Zukünftig könnten sensorbasierte Systeme Echtzeitdaten liefern, um Erst- und Folgeanpassungen noch besser an veränderte Bedingungen anzupassen.

### Profilerhebungen zur präzisen Bedarfsermittlung

Profilerhebungen ergänzen medizinische Untersuchungen durch funktionelle Tests und Bewegungsanalysen, die spezifische Anforderungen an Hilfsmittel definieren. Technologien wie Videotechnik und Sensoren liefern Daten zu Gang, Haltung und Gleichgewicht. Sie unterstützen die Hilfsmittelauswahl und ermöglichen iterative Anpassungen. Zukünftig könnten tragbare Sensoren und Analysen mithilfe der künstlichen Intelligenz (KI) die Versorgungsqualität steigern und standardmäßig eingesetzt werden, um Versorgung und Forschung gleichermaßen zu verbessern.

### Assessments zur objektiven Bewertung des Therapieerfolgs

Assessments ermöglichen eine objektive Bewertung von Therapieerfolg und Versorgungsbedürfnissen durch standardisierte Fragebogen und Tests. Sie erfassen Patientenerfahrungen wie Zufriedenheit, Schmerz und Alltagstauglichkeit und unterstützen die kontinuierliche Optimierung der Hilfsmittel [1].

Zukünftig könnten digitale Fragebogen und Apps Patienten ermöglichen, regelmäßige Feedbacks zu geben, wodurch Versorger schneller Anpassungen vornehmen können. Diese Technologien verkürzen Anpassungszyklen und verbessern die Dokumentation und Analyse von Therapieerfolgen.

### Regulatorische Anforderungen und ihre Bedeutung für klinische Studien

Die europäische MDR verlangt beispielsweise gemäß den Kapiteln VI und VII hohe Sicherheits- und Wirksamkeitsnachweise für Prothesen und Orthesen, unterstützt durch klinische Studien und Daten. Klinische Untersuchungen, Profilerhebungen und Assessments werden künftig, insbesondere bei Sonderanfertigungen, entscheidend sein. Sie fördern standardisierte Bewertungsprozesse und liefern Daten für Zertifizierungen, Marktbeobachtungen und Produktverbesserungen, wodurch Trends und Muster zur kontinuierlichen Qualitätssteigerung erkannt werden können. Damit können, beispielhaft genannt, auch die wichtigen Anforderungen der MDR gemäß Kapitel VII an die Überwachung und Vigilanz erfüllt werden.

### Klinische Anforderungen

Klinische Untersuchungen, Profilerhebungen und Assessments sind zentral für eine personalisierte Versorgung. Sie verbessern die Lebensqualität der Patienten und erfüllen regulatorische Anforderungen. Zusätzlich erfassen sie differenziert Bedürfnisse und Fähigkeiten, ermöglichen individuelle Anpassungen und unterstützen die Bewertung des Therapieerfolgs. Zukünftige Technologien wie tragbare Sensoren, Echtzeitanalysen und digitale Feedback-Systeme werden die Hilfsmittelanpassung weiter optimieren sowie deren Sicherheit und Wirksamkeit steigern.

### Verstärkte Patientenbeteiligung für ein „patient empowerment“

„Shared decision-making“ und eine informierte Einwilligung fördern die Zusammenarbeit bei Behandlungsentscheidungen, steigern die Patientenzufriedenheit und reduzieren Doppelversorgungen sowie unnötige Kosten. Betroffenen fehlen oft Kenntnisse über Behandlungsmöglichkeiten und Versorgungsabläufe, während sie als Informationsvermittler zwischen Fachrichtungen agieren und einen zentralen Ansprechpartner vermissen [12]. Transparenz im Versorgungsprozess, etwa bei Rehabilitationszielen, stärkt Betroffene. Studien zeigen, dass ausreichende Patienteninformation und -einbindung die Prothesenakzeptanz, Lebensqualität und Zufriedenheit der Betroffenen verbessern [6].

## Fazit für die Praxis

- Das Register erlaubt, relevante Daten strukturiert zu erfassen, zu verwalten und für Analysen zur Verfügung zu stellen.
- Das AMP-Register bietet eine wertvolle Grundlage für die Identifikation von Versorgungslücken und erfolgreichen Behandlungskonzepten.
- Das Register erlaubt eine objektive Bewertung der Mobilität und gesellschaftlichen Teilhabe von Betroffenen.
- Durch die Nutzung standardisierter Daten können klinische Bewertungen und Versorgungskonzepte langfristig optimiert werden.
- Das Register fördert die Vernetzung aller beteiligten Berufsgruppen und ermöglicht eine objektive Bewertung der Patientenentwicklung.
